# British Asylums for the Insane

**Published:** 1855-01-01

**Authors:** 


### THE JOURNAL

OF

### PSYCHOLOGICAL MEDICINE

AND

### MENTAL PATHOLOGY.

JANUARY 1, 1855.

### Art. I.—BRITISH ASYLUMS FOR THE INSANE.

We propose inaugurating our new volume by placing before our readers an analysis of the most recent reports of the British County Asylums. These annual records contain a vast body of useful general and statistical information in reference to the condition of the pauper portion of our insane population. These reports are not easy of access to many of our foreign subscribers, and it becomes, therefore, important that we should annually publish a fair *résumé* of their contents. The official documents now under review are evidently drawn up with great care. The tabular statements embodied in the reports must have entailed upon the medical officers much thought and laborious work. Without further preface, we now proceed to an analysis of the "Ninth Report of the Hanwell County Lunatic Asylum for 1854:"—

"The number of patients admitted during the year was 126, of whom 64 were males, and 62 females. The number discharged cured was 43, of whom 17 were males, and 26 females. Three males also left the Asylum improved. The number of deaths was 68, of whom 45 were males, and 23 females. The rate of mortality, though somewhat higher than last year, is not above the average."

Dr. Begley says—

"The number of patients in the male division of the asylum at the commencement of the year was 411; 64 have been admitted since, making together 475; 17 have been discharged cured, 3 as improved, and 45 have died, leaving 410 now under care.

"The patients admitted during the year were generally in so chronic a stage of their disorder, or the malady was so much complicated with general paralysis or epilepsy, diseases which usually render insanity incurable, that hope could not be entertained of the recovery of many of them. Some of those, however, who were received during the early stage of the disease, and who were not affected with either of the complications referred to, have left the asylum well; others of them are progressing favourably, and are likely to be discharged cured in the ensuing spring.

"In 38 of the cases admitted the disease assumed the form of mania, and in 9 that of melancholia. Imbecility was the type of the disorder in 15 cases, and dementia was manifested in 2. Mania was associated in 6 cases with general paralysis, and in 6 others with epilepsy. There was a double combination of general paralysis and epilepsy in 1 case of mania. Melancholia was found in union with general paralysis in 1 case. Imbecility was combined with that affection in 8 cases, and with epilepsy in 1; in 1 other case of imbecility there was a combination of both paralysis and epilepsy. The 2 cases of dementia were complicated with general paralysis.

"A tendency to suicide was united with 1 case of mania, and with melancholia in 7 cases; 1 of the suicidal cases of melancholia was affected with general paralysis."

Dr. Begley refers to the cases of three patients who were discharged "cured," after being 11, 4, and 6 years inmates of the asylum. He justly observes, that "recoveries like these 3, after so long a residence in the Institution, are occasionally met with in every asylum, and tend to show that hope is not to be abandoned in any case, however unpromising or protracted."

The deaths were, as usual, "caused by general paralysis, apoplexy, epilepsy, and general debility; some others resulted from dropsy, diseases of the heart and lungs, stomach and bladder."

Mr. Denne, the medical superintendent of the female department, has made but a short report. He says—

"On the first of January, 1853, there were 552 female patients in the asylum.

"The average number resident during the year has been 557.

"Sixty-three patients have been admitted; about half of whom, from either age, infirmity, or advanced stage of disease, must be added to the list of incurables.

"Twenty-three patients have died; of whom 2 were upwards of 90 years of age, 5 above 80, 2 above 70, 8 above 60, and 6 between 20 and 50, the average of the whole number being 61½ years."

The Chaplain's report is satisfactory. The majority of the patients manifest an eagerness to be present at divine service. At the last celebration of the holy communion in the asylum, 55 patients attended! The following case forcibly illustrates the importance of bringing the insane within the soothing influences of religion:—

"On entering the wards after the evening service, a patient came to meet me, and said, 'I thank you, sir, very much for your sermon to-day, it has taken a load, as it were, from my mind, for I feared I should not be able to resist the dreadful thoughts I have lately had; but now I believe, through God's help, that I shall be able to get rid of them.' On my remarking, 'But you were not at chapel, N.,' he replied, 'No, I was too ill to sit so long, but I stood outside the door and listened.' Surely in this we have an example of the Word being blessed, 'to strengthen the weak hands and confirm the feeble knees.'"

The Hanwell committee have thought proper to publish a report from the Matron of the asylum. Surely this is unnecessary? It places her in a false position, and is, we think, derogatory to the dignity, and detrimental to the usefulness of the medical staff. There is nothing in her report that entitles it to this honour. It is entirely devoid of interest: the facts she records are of no practical importance or value. In making these observations, we repudiate all intention of depreciating the talents, activity, industry, and humanity of Mrs. Macfie; but we would advise her for the future to leave to the medical officers the duty of doing the literary work of the asylum. She must have much to occupy her time in discharging faithfully the onerous duties that specially devolve upon one holding her responsible post. In writing accounts of cases in the asylum for publication in the annual report she is evidently out of her element. The committee of Hanwell Asylum will do well to omit, for the future, this kind of addenda.

The report contains several valuable tables well worthy of attention.

The "Third Annual Report of the County Asylum at Colney Hatch" next merits attention. It appears that—

"During the past year, 392 patients have been admitted,—namely, 254 males and 138 females. The deaths during the spring were more than the average, on the male side; but during the year the number has not been more than might have been expected, considering the state in which many are sent to the asylum.

"On the female side, the mortality has not been greater than in former years.

"The total number of deaths is 208,—namely, 135 males and 73 females.

"The discharges of patients recovered during the year have been—100 males and 42 females—a number which, if not equal to our benevolent desires, is beyond what might have been expected, and exceeds that of last year by 10 cases."

Mr. Tyerman, head of the male department, records the following statistics:—

| | | |
|---|---|---|
| There remained in the male department, 31st Dec., 1852 | 514 | |
| There have been admitted since ... ... ... ... ... ... | 254 | |
| Total males under care during the year ... ... ... | —— | 768 |
| There have been discharged— | | |
| Recovered ... ... ... ... ... ... ... ... ... ... | 100 | |
| Relieved ... ... ... ... ... ... ... ... ... ... | 23 | |
| Unrelieved ... ... ... ... ... ... ... ... ... | 6 | |
| Died ... ... ... ... ... ... ... ... ... ... ... | 134 | |
| Total discharged and dead ... ... ... ... ... | —— | 263 |
| Remaining under care Dec. 31st, 1853 ... ... ... | | 505 |
| Daily average number ... ... ... ... ... 509 | | |

In speaking of the epileptic cases, Mr. Tyerman says that "10,000 epileptic fits occur annually among the male patients alone."

It appears that—

"*Post mortem* examinations have been instituted in 104 cases, the results proving very generally the long previous existence of organic affections; pulmonary disease, tubercle, &c., was associated in 89 instances, heart disease in 51, and kidney disease in 42. Abnormal conformation of the brain has been occasionally observed; *e.g.* want of symmetry between the hemispheres and central portions, and in rare instances the posterior conua of the lateral ventricles were not developed."

Mr. Marshall, medical superintendent of the female department of the asylum, says,—

"In consequence of the house being nearly full at the end of the year 1852, the admissions of patients have not been quite so numerous, amounting to 138, whilst during the year, 42 have been discharged as recovered, 16 relieved, and 8 not improved, have been removed to other asylums, and 73 have died, leaving 728 patients under treatment in the asylum on December 31, 1853, vacancies for 8 persons only remaining in the various wards of this department of your asylum."

This gentleman appends to his report several interesting tabular statements. The account of the *post mortem* examinations appear to be clearly and ably drawn up, and do credit to the medical officers of this national establishment. From Colney Hatch Asylum we proceed to another great public institution, the annual report of which is now before us. We allude to Bethlem Hospital. We have been much pleased with Dr. Hood's account of the state of this asylum. His report is drawn up with ability, and embodies many facts of deep interest to the psychological physician. First, as to the statistics of Bethlem, Dr. Hood says,—

"On the 1st of January, 1853, there were 356 patients (including those out on leave) in the hospital, of whom 194 were males, and 162 emales; and during the year 242 patients were admitted—105 males, and 137 females; so that since the last annual report 598 patients have been under medical treatment and moral surveillance.

"The admissions were as follows:—

| | M. | F. | Total. |
|---|---|---|---|
| "Curable ... ... ... ... ... ... | 72 | 128 | 200 |
| Incurable ... ... ... ... ... ... | 1 | 3 | 4 |
| Criminal ... ... ... ... ... ... | 32 | 6 | 38 |
| | 105 | 137 | 242 |

"The number of admissions in this, and in all other public institutions, is subject to remarkable fluctuations: thus, in the year 1849 there were received into this hospital 344 patients; the next year the number rose to 374, and in the following year, viz. 1851, the admissions fell to 306.

"It is, therefore, neither surprising nor disparaging to find the number of admissions last year was less than during preceding years, which was reasonably accounted for by my predecessors, who observed, in their report addressed to you in 1851, that 'this diminution of admissions may be ascribed to the numerous county asylums which have been erected in different parts of the kingdom, and which must necessarily diminish in a material degree the applications for admission into this and similar institutions.' We have also to consider that in the metropolitan district alone competition has induced the proprietors of some private asylums to adopt such a reduction of terms as render many of these establishments available to the middle classes of society—persons on the verge of poverty, who would otherwise require charitable assistance. Among the admissions you will observe 38 criminal lunatics—32 males and 6 females—being an increase of 10 upon the number admitted in 1852, and of as many as 24 upon the number admitted in 1851.

"The discharges and removals of patients during the year amounted to 212, of whom 82 were males, and 130 females; of these numbers it is satisfactory to state that 121 were dismissed cured. The details are as follows:—

| | M. | F. | Total. |
|---|---|---|---|
| "Cured ... ... ... ... ... ... | 45 | 76 | 121 |
| Uncured ... ... ... ... ... ... | 35 | 48 | 83 |
| Removed at request of friends | 2 | 6 | 8 |
| | 82 | 130 | 212 |

"The peculiar constitution of this hospital, which by its regulations restricts the period of a patient's residence on the curable establishment to one year, with a discretionary extension to three or six months—which was wisely devised with the view of making it a strictly curative institution—may explain the reason of so many patients being discharged *uncured.* The majority of them were in fact still under medical treatment, though with little if any hope of permanent amendment: it being well known that the chances of recovery are materially diminished after the first year; and the majority of cures here reported will be found to have taken place within the first three months after admission.

"The deaths which took place during the year, it is highly satisfactory to state, were not so numerous as I had reason to anticipate in my last report."

When referring to the adoption of the "Non-restraint System," Dr. Hood gives some interesting details of the barbarous mode of treatment pursued towards the insane during the "dark ages." We are glad to perceive that Dr. Hood is directing his attention to moral treatment, and that he is disposed to carry out in a liberal and enlightened spirit the curative principle acted upon with such great success in the majority of the best first-class private asylums in this country. He justly observes,—

"In the moral management of the insane we cannot attach too much importance to those occupations and recreations which tend to divert the mind from its delusions, and which rouse and invigorate the healthy exercise of its reflecting faculties. Speaking generally, we find lunatics of every class, unless urged to the contrary, disposed to be indolent; some few indeed may be mischievously restless: but the majority succumb under their morbid feelings, and are indisposed to exert themselves with any degree of steadiness.

"There can be no doubt that every description of occupation has a curative tendency, and it is desirable that such patients should receive every possible encouragement. Even recreation, whatever be the kind of amusement, is only another term for mental employment, and judiciously promoted, cheers the mind, and excites a healthy tone of feeling. Hence some of the patients during the year were permitted to walk out, under the care of nurses and proper attendants, which was esteemed a great indulgence, and had perceptibly a good effect. Four of the male patients, who were, however, not fit to be discharged, were allowed to spend a day at Kew, another day they went by steam-boat to the Nore; and conducting themselves well, under the charge of careful attendants, during the year visited many different public exhibitions, the National Gallery, the Crystal Palace, Marlborough House, the Zoological Gardens, Smithfield Cattle Show, &c. &c. This privilege was awarded to them gradually, and was suggested by their enjoyment and quiet demeanour when first taken for a walk round the garden: and I have no hesitation in stating that this indulgence having been highly appreciated by them, has had a beneficial effect upon their minds. If we can succeed in giving a patient the impression that we repose confidence in him, if we can make him sensible of the importance of keeping his *parole d'honneur*, we are greatly improving his mental state: for the recovery of self-respect is often the first indication of impending cure. Hence we find the reports of many lunatic asylums attesting the advantages which patients derive from such excursions."

We regret that our limited space prevents our quoting more at length from this valuable report. We can specially commend it to the notice of our readers. The tabular statements deserve careful study.

The following is the statistical statement of the medical department of the Surrey Lunatic Asylum, for 1853, published in the report for 1854:—

"At the date of our last report there were 396 male patients, and 488 female patients—together 884. Since then, there have been admitted 136 males, and 193 females—together 329; and 342 have been discharged, or died, leaving, at the close of the year, 871.

"The total number of patients in the asylum, during the year, was 1213; the highest number at any time was 911, the lowest was 864; and the average number under treatment, during the whole period, was 887.

| | M. | F. | Total. |
|---|---|---|---|
| "Remaining 31st December, 1852 ... ... | 396 | 488 | 884 |
| Admitted in 1853 ... ... ... ... ... | 136 | 193 | 329 |
| | 532 | 681 | 1213 |

"Of whom have been discharged—

| | M. | F. | Total. | | | |
|---|---|---|---|---|---|---|
| Recovered ... ... ... ... ... ... | 65 | 104 | 169 | | | |
| Removed, not recovered ... ... | 26 | 30 | 56 | | | |
| Died ... ... ... ... ... ... ... | 65 | 52 | 117 | | | |
| | | | | 156 | 186 | 342 |
| | | | | 376 | 495 | 871 |

"The number of recoveries is nearly the same as last year, being in the proportion of about 14 per cent.; that of deaths is rather greater, being about 9 per cent.

"Of the deaths of 65 male patients—

27 died of apoplexy and general paralysis.
13 " exhaustion, general debility, and old age.
7 " epilepsy.
8 " pulmonary disease.
10 " of various other causes.

"Of the 52 female patients—

17 died of apoplexy, cerebral disease, and general paralysis.
9 " pulmonary disease.
7 " epilepsy.
7 " exhaustion.
3 " old age.
7 " of various causes.

"Three cases came under the investigation of the coroner and a jury, whose verdicts were as follows:—

"In one case, a male, 'natural death—sudden exhaustion upon excitement, after an attack of delirium tremens.'

"In one case, a male, 'suicide, by thrusting a portion of a glove into the throat, producing suffocation—he being insane.'

"In one case, a female, 'natural death, from apoplexy.'"

With the exception of the tables, the report contains no facts or remarks calling for particular notice. Sir A. Morison, whom we believe prepares the tabular statements of this asylum, deserves much praise for the ability with which he conducts this department of the asylum.

From the "First Annual Report of the Norfolk County Lunatic Asylum" we ascertain that—

"During the past twelve months, 83 patients have been admitted; viz., 36 males and 47 females.

"On the 31st December, 1852, there were in the asylum, 139 males and 159 females; total, 298. The whole number under treatment during the year has been 381; the average number daily, resident, 304.91, or 139.66 males and 165.25 females.

"The number of deaths has been 36, or 19 males and 17 females; and the number of recoveries 39, or 17 males and 22 females; and the number discharged, not cured, has been 6; viz., 2 males and 4 females."

The report, which is drawn up by Dr. Foote and Mr. Firth, is entitled to our warmest commendation. We regret that Dr. Foote should have been removed from this sphere of usefulness, and sincerely hope that, ere long, he will be again in harness, and busily engaged among the insane in the work of labour and love.

We extract from the "Second Annual Report of the Derbyshire County Lunatic Asylum, for 1854," under the able management of Dr. Hitchman, the subjoined interesting particulars:—

"It is a remarkable coincidence that the admissions during the past year are almost numerically the same as those of the year 1852. From January, 1851, to January, 1852, there were admitted into this asylum, 73 male and 57 female patients, and during the year that has just passed, there were admitted,—

| | | |
|---|---|---|
| Males ... ... ... ... ... | 74 | 131 |
| Females ... ... ... ... | 57 | |

being the precise number of females, and only one in excess of the number of males admitted during the corresponding period of 1852.

"There were more patients admitted in the month of June than in any other month of the past year—the largest number of admissions being 17 in June, 16 in April, and 13 in September. As, however, only 4 out of the 17 were of recent origin, this fact does not throw much light upon the influence of seasons in the production of the malady. The largest number of recent cases were brought to the asylum during the month of January; whether an equal number of cases sprung up in any other month of the year, it is not possible to state, as there is much reason to fear that patients are still kept back from the institution by financial and other considerations.

"In those countries where cretinism and idiotism are endemic, it has been found that the greater number of cretins were resident on the declivity of the mountains towards the north. This fact has been proved by the Sardinian Commission, and by the private researches of scientific men, but has been most specially enforced by Dr. J. C. Hubertz, of Copenhagen. This observer has also found that insanity in Denmark is more prevalent in the northern 'herreds,' or divisions of the kingdom, than in the southern portions. All the facts which have hitherto fallen under the observation of your physician would appear to substantiate this conclusion, as far as idiotcy is involved in this county, but it does not appear to hold good in reference to the number of the insane in the two divisions of Derbyshire. Of 258 patients sent from this county since 1851, 92 were from the northern division, and 166 from the southern portion, in which the town of Derby is included. The population of the two divisions being—

| | |
|---|---|
| Northern Division ... ... | 130,067 |
| Southern Division ... ... | 166,017 |

the proportion of the insane to their respective populations will be—

Northern Division ... 1 in every 1413 $\frac{71}{92}$

Southern Division ... 1 in every 1000 $\frac{17}{166}$

Or, leaving Derby out of the calculation, which in common with all large towns has special influences in operation upon the human mind and feelings—the statistics would be as follow:—96 from the southern division, containing a population of 125,408, making a proportion of 1 in every 1306$\frac{1}{3}$. This would give a slight preponderance in favour of the mental salubrity of the northern division; as the parliamentary division has been taken in this calculation, some villages are included in the southern division which are, in truth, more northerly in their geographical position than those which are enumerated as northern,—for instance, Matlock in contrast with Alfreton, or Pinxton; but the population is not sufficiently large to affect in any essential degree the above conclusions. From the town of Derby 70 patients were sent, which in a population of 40,609 give a proportion of 1 in every 580$\frac{9}{70}$.

"Even if the whole island be taken into calculation, we observe no especial exemption in favour of the southern counties. Dorset, one of the most southerly, abounds in lunatics and idiots, ranging as high as 1 in 640 of the population, while Derbyshire, Durham, and Lancashire, according to the Poor-law returns, have not above 1 lunatic or idiot in 1000 of the population. Thus teaching us that there are other causes as potent in the production or prevention of nervous diseases as geographical position.

"The social condition and occupations of the patients admitted during the year were various, as shown by the following tables:—

| Males. | | Females. | |
|---|---|---|---|
| Single ... ... ... | 31 | Single ... ... ... | 30 |
| Married ... ... | 38 | Married ... ... | 21 |
| Widowed ... ... | 4 | Widowed ... ... | 6 |
| Unknown ... ... | 1 | | |
| | 74 | | 57" |

When speaking of the effects of religious excitement in producing insanity, Dr. Hitchman observes,—

"All impassioned religious excitement which does not culminate in some useful act, has a tendency to agitate and overwhelm weak and sensitive persons, and that the peculiar dogmas embraced are largely determined by circumstances, and by inherent and special characteristics of the individual mind; and that it is unjust to charge upon any special religious theory the fearful consequences ascribed to the Calvinistic creed. Minds linked to a special organization become excited and bewildered by the stern, exclusive, and yet impassioned tenets of the Geneva Reformer; but then minds of another character become equally disturbed by the more diffusive creed of Wesley and his followers. Indeed, during the early career of John Wesley, it is certain that more persons became convulsed, and ultimately insane, than during the preaching of George Whitefield. Southey informs us, when speaking of the convulsions which agitated many of Wesley's followers, that 'These effects had never as yet been produced under Whitefield's preaching, though they now followed Wesley wherever he went; and it appears that Whitefield, who came once more to Bristol at this time, considered them as doubtful indications at least, and by no means to be encouraged. But no sooner had *he* begun to preach before a congregation, among whom these 'outward signs' had *previously* taken place, and who therefore were prepared for the affection by their state of mind, as fear in times of pestilence predisposes the body for receiving the contagion, the four persons were seized almost at the same moment, and sunk down close by him. (Southey's 'Life of Wesley,' p. 281.) 'According to a moderate computation four thousand people were within a very short time affected with this convulsive malady.'—Hecker on the 'Dancing Mania,' p. 134. These susceptibilities are dependent rather upon constitutional peculiarities than upon the effect of special tenets; thus we have perceived in the history of individuals, that even the Holy Scriptures may breathe a solace and a peace to one individual, and yet arouse, as if with the tones of a trumpet, the combativeness and energy of another. 'Scripture,' said Melancthon, 'imparts to the soul a holy and marvellous delight, it is the heavenly ambrosia.' 'The Word of God,' exclaimed Luther, 'is a sword, a war, a destruction; it falls upon the children of Ephraim like a lioness in the forest.' The excess of *fanaticism*, its immoderate ecstasies, and selfish raptures agitate the nervous system, disorder its functions, and bring the reason and the will under the sole dominion of imagination and feeling, and thus occasionally render the individual insane and irresponsible, both in our own church or in the church of the Vatican, as in the wildest of the sects which spring up in this and other countries; but where one man now falls a victim to 'religious excitement,' ten others are the prey of exhausting anxieties contingent upon commercial affairs—the fatigues of overwork—or of vicious indulgence in forbidden pleasures; whilst to many in asylums, as to thousands in the world, the religion of the Gospel has been 'a message of glad tidings,' and a balm, a consolation, and a peace more sustaining and restorative than any other single agency."

Dr. Hitchman says, when speaking of the causes of insanity,—

"In a very large proportion of the cases the malady was hereditary—and on more than one occasion during the past year, two and three members of the same family have been under treatment at the same time. Intemperance, domestic trials, disappointed affections, and bodily ailments of a special kind have been the other most clearly ascertained causes, and their frequency has been in the order in which they have been enumerated."

Again, when referring to the effect of "anxiety" and "mental shock" in disturbing the functions of the brain, Dr. H. observes,—

"Emotion and shock are far more frequently the cause of insanity than prolonged intellectual exertions of any kind. Intellectual labour rarely disorders the mind permanently, unless *anxiety* or some other powerful emotion is superadded to it. The student ambitious of distinction and fearful of defeat—the merchant harassed by business, and dreading an altered position in his circumstances, or the discredit and disgrace of bankruptcy—the devotee perplexing himself with conflicting creeds, anxious to be of the true church and yet distrustful of his previous convictions—the fanatic yielding himself up to wild and rapturous emotions—the mechanic or labourer toiling too much under the anxieties of home and family—are all engaged in dangerous pursuits, which have wrecked and will continue to wreck many minds, no matter what may be the peculiar study, the especial business, the particular creed, the special sect, or the kind of labour which may be engaging their respective attentions. In carefully investigating the histories of patients, it has been usually found that physical weakness and moral shock have combined to produce the unhappy result."

It is undoubtedly true that there exists an essential distinction between the pathological effects of pure intellectual exertion and emotional influence; but we do not agree entirely with our author, that "intellectual labour rarely disorders the mind permanently." We have seen *many* distressing cases of incurable insanity, which would clearly be traced to "intellectual labour." Softening of the brain and permanent impairment of the intellect, ending in general paralysis, according to our experience, are often the consequences of excessive devotion to literary and intellectual pursuits. We admit, however, the difficulty of disassociating severe intellectual work from anxiety of mind; they appear to us very often to proceed *pari passu*. When speaking of the medical treatment of insanity, Dr. Hitchman observes,—

"In cases of aggravated hysteria associated with corporeal debility, the tincture of sumbul combined with Battley's sedative, has been a most useful medicine, and especially when the milder preparations of iron, such as the citrate, have been administered in the interval."

When referring to the question of "Non-restraint," our author remarks,—

"No less than forty-seven suicidal individuals have been under treatment, and some of these have been so energetically bent upon effecting their purpose, that it was impossible to leave them for day or night for many weeks in succession. A few of them involved great responsibility, and formed, indeed, these especial cases which are said to test and to refute the principle of non-restraint. The opponents of this practice are constantly asking—'What would you do with a man who had cut his throat and was determined to pull it open again after it had been dressed?' Indeed, all kinds of imaginary cases are conjured up, to which you are expected to give an explicit and categorical reply. But no treatment can be defined for such ideal cases, and no defined treatment will succeed (because never *heartily* carried out) in the hands of an unbeliever in the efficacy and humanity of the principle."

We should be disposed, with deference to Dr. Hitchman, to repeat the question he has put into the mouth of "the opponents of this practice," and ask, how *he* would treat a case of this kind? We cannot conceive why Dr. Hitchman should call such cases "imaginary" or "*ideal*," when they are of ordinary occurrence. Lunatics do, alas! sometimes cut their throats—ligatures are occasionally required for the treatment of these cases; and patients resolutely determined upon self-destruction often do their utmost to effect their purpose, by tearing the wound open! Under such distressing circumstances, who would for one moment question the necessity and humanity of preserving life, by restraining the hands of the patient until the wound has cicatrised?

We find nothing in the "Fifty-eighth Annual Report of the Friends' Retreat for 1854," calling for special comment. We can only afford space for one extract. Mr. John Kitching, the resident medical officer, when speaking of the treatment of the insane, justly remarks:—

"That mode of treatment approaches most nearly to our idea of perfection, which adapts itself to the specialities, physical and moral, of each case, and seeks to maintain each individual in the highest condition of which his mental faculties are capable. Kindliness and sympathy for suffering and misfortune form the foundation-stone on which all effectual treatment is based. It may, however, happen that the kindest treatment requires a very different plan to be pursued from what the patient may approve, and he may form a very mistaken estimate of that which is really the most conducive to his welfare; to him the greatest kindness may put on the disguise of its opposite, and the conductors of asylums must be prepared to be misunderstood and misrepresented in much that they do with the single-hearted desire to promote the interests of those under their charge."

There are several valuable tabular statements appended to the report, to which we can only direct attention. We glean the following statistical facts from the "Third Annual Report of the Medical Superintendent (Dr. Thurnam) of the Wilts County Asylum":—

"At the beginning of the year which has now expired, there were in the asylum 219 patients; namely, 103 males and 116 females. In the course of the twelve months, there have been 105 cases admitted; of which 44 were males and 61 females:—the average being almost precisely at the rate of two cases per week. Of the whole number of admissions 18 were persons who had previously been discharged, or cases of re-admission. There are now in the asylum 255 patients; namely, 110 males and 145 females. The average number resident during the year has been 244·6.

"There have been 46 discharges; and of this number, 34 were considered as recovered when they left the asylum. The majority of the remainder were much improved in their mental condition, and were for the most part discharged at the request of their friends. A few were removed to other asylums, as not chargeable to parishes in Wiltshire. Two patients are absent on trial.

"Of the whole number discharged since the opening of the asylum, 19 cases have been re-admitted during the year, in consequence of a relapse or recurrence of the disorder."

Dr. Thurnam, when referring to the liability to relapse in cases of apparent recovery from attacks of insanity, observes—

"Many also, who whilst subject to the regular discipline of an asylum, appear well and fully capable of self-government, are no sooner at liberty than they display symptoms of the disorder, which had been restrained or concealed, rather than eradicated or cured."

According to our experience, such is often the case. It is said that Zimmerman, the author of the celebrated work on "Solitude," was never sane outside of the walls of a lunatic asylum, but became rational and free from excitement soon after being placed under restraint; but immediately upon his release, relapsed into his former condition.

The statistics of the "Lincolnshire County Lunatic Asylum" for the past year are conveyed in the following paragraphs, taken from the first annual report. This asylum, we would premise, was opened for the reception of patients on the 9th of August, 1852:—

"On the 31st of December, 1853, the number of patients who had been admitted was as follows, viz.:—84 men and 92 women from other asylums; 8 men and 12 women from workhouses; and 64 men and 57 women from their homes; making a total of 317 patients, of whom 156 were men and 161 women.

"Seventeen men and 18 women were discharged recovered; 5 men and 3 women (out-county patients) were removed to other asylums; and 19 men and 14 women died; making the total of discharges and deaths 76—41 men and 35 women.

"The numbers remaining in the asylum on the 1st of January, 1854, were 115 men and 126 women; total, 241.

"The average daily resident number for the year 1853 was 228.23; and for the whole period of seventeen months, 205.81.

"The mortality was 10.41 per cent. of the whole number under treatment, and 16.03 per cent. of the mean resident number; which, regarding the infirmities and bad bodily condition of a large number of the patients when admitted, is by no means an unfavourable result."

Nothing can be more just than the following remarks on the importance of early and prompt treatment:—

"In cases of recent occurrence, however, the importance of early removal cannot be too strongly urged. All experience and all authority assert that when once insanity is manifested, the very foundation of curative treatment consists in removing the patient from the external influences which have occasioned or are likely to protract the disorder, in overcoming resistance to remedial agents, and in adopting an appropriate regimen and diet. These conditions of treatment, so far at least as the indigent classes are concerned, can only be complied with by removal to an asylum; and any delay in effecting this, whether from false economical motives on the part of parish authorities, or from repugnance of friends to the separation, is fraught with injury to the patient and ultimate expense to the ratepayers. A week's procrastination may protract the treatment to months; a month's delay may allow a favourable crisis to pass by unimproved, and determine the chronic stage of the disease."

When alluding to the previous occupations of the patients admitted, as well as the hereditary character of the insanity, it is observed—

"As would be expected in a county whose population is essentially agricultural, the admissions have included a large number of farm-labourers, their wives and families. From the healthful and unexciting nature of its employment, it might be supposed that such a population should enjoy a larger immunity from insanity than that of manufacturing counties, but such does not appear to be always the case. The proportion of insane paupers to the population of Lancashire is as 1 in 1083, in the West Riding of Yorkshire as 1 to 1176, and in Staffordshire as 1 to 1079; while in Lincolnshire it is as 1 to 806—a proportion which, it is believed, is largely attributable to hereditary predisposition. In many of the cases received from the towns the mental disorder has been distinctly traceable to habits of intemperance and dissipation; but in those coming from the rural districts of the county such causes have been comparatively rare, and a congenital want of mental power to resist ordinary excitants and depressants has appeared pretty generally to have been the *fons et origo mali.*"

A high authority has declared that the stomach-pump is never necessary in the treatment of the insane. What would, we ask, have become of Dr. Palmer's patient if he had not forced food into the stomach by means of this instrument?

"A few instances of refusal of food have occurred, but, with the exception of one female patient, yielded to change of diet and medical treatment. The patient alluded to fell, soon after her admission, into a cataleptic state, during which no inducement whatever succeeded in getting her to swallow anything. If her mouth was opened, and food put into it, she would remain with the food resting on her tongue until somebody removed it. After several days' abstinence her strength began to fail, and the odour of her breath indicated that feeding could be no longer delayed with safety. The stomach-pump was employed three times a-day, without her offering the least resistance, for six weeks, when her health became much improved, and she began to eat again of her own accord. She subsequently mended very rapidly, and has since been discharged quite recovered."

There appear to have been four inquests at the asylum; one patient died during a fit of epilepsy; the second died suddenly from ulceration and perforation in the upper portion of the intestinal canal; the third was a case of suicide from hanging; and the fourth case was "also a male patient, who died in consequence of his ribs having been severely injured during a paroxysm of maniacal violence, and while two of the attendants were conveying him down a flight of stairs for the purpose of placing him in a padded-room. The verdict returned by the jury was 'homicide by misadventure.' The whole of the circumstances attending the case were subsequently investigated at a special meeting of the visitors, who were of opinion that the verdict of the inquest was entirely supported by the facts."

We do not affirm this accident could have been averted if the strait-waistcoat had been at once applied; but of this we feel *strongly convinced*, that it is much more humane to apply such restraint for a short period than for four or five powerful men to struggle with a patient in "a paroxysm of maniacal violence." Severe and serious injuries may and do often occur from these absurd contests with the insane.

Dr. Palmer's report does him great credit. We congratulate the committee on having so active and intelligent an officer at the head of the Lincolnshire Asylum.

According to the "Eighth Annual Report of the Medical Superintendent of the Devon County Lunatic Asylum, for 1850," there were admitted—

"During the past year, 96 patients, of whom 49 were men and 47 were women. The largest number resident at one time was 472; the number under treatment has been 555; and the average number resident has been 460.

"The number of patients at the commencement of the year was 459; and the number resident at this date is 445, of whom 195 are men and 250 are women.

"Sixty-two patients have been discharged, of whom 26 were men and 36 women; of these 55 were discharged recovered—6 were discharged relieved—and 1 unimproved.

"Forty-seven patients have died, of whom 25 were men and 22 were women.

"The mortality which last year was 6.6 per cent. of the average number resident, has this year been 10 per cent. of that number, and 8.4 per cent. of the number under treatment.

"Many of the patients whose deaths have this year swelled the obituary, were admitted in a dying state. No. 1072, a melancholic, was unable to retain any food after admission, and died in twenty-six days, from disorganization of the stomach. The appearance of this organ suggested the probability that the unhappy man had taken some deleterious substance before admission, with a suicidal intent.

"No. 1099 was admitted in a state of extreme exhaustion, from general paralysis, with mania, and sank twenty-two days afterwards from decay of the powers of nature. No. 1151, who died in six days after admission, was a similar case. No. 1100, a miner, was admitted with both lungs in a state of disorganization from that form of consumption, known as coal miners' lung; he survived about four months. No. 1110 survived nearly as long; he had sustained two attacks of apoplexy, and was suffering, on admission, from double hemiplegia, which paralysed both sides of the body, and left him in the most perfect state of wreck—mental and bodily. No. 1163, who had been insane three years, (but had only recently become chargeable), was admitted in a dying state, with one lung and part of the other in a state of decomposition, from pneumonia; he survived sixteen days. No. 1168, admitted with inflammation of the intestinal canal, survived six days. With the exception of the last, who was 68 years old, the above patients were of middle age.

When speaking of medical and moral treatment of the cases confided to his care, Dr. Bucknill observes,—

"During the past year no efforts have been neglected which were requisite to maintain the medical treatment of the patients in proximity with the ever-advancing steps of medical science. Among the novelties of treatment it may be mentioned that epilepsy has been relieved in the most satisfactory manner by tracheotomy; that dementia has been relieved by phosphuretted oil; that chorea with mania, threatening a fatal termination, has been cured by the internal administration of chloroform; and that extreme excitement, in which other remedies had failed, has been removed by frequent small inhalations of the same remedy. Of these results some have already been communicated in detail to the profession, and others will be so through more appropriate channels than this general report.

"The same system of moral treatment mentioned in former reports has been continued with satisfactory results in the recovery of those patients whose malady was capable of cure; and in the reform of bad habits, the amelioration of symptoms, the increased quietude, comfort, and happiness of those whose malady is irremovable, and for whom the asylum must be considered a permanent home.

"During nine months of the year (and in all the wards except those occupied by the idiots) the evening reading classes are kept up with benefit and punctuality four evenings in every week. Of the other three, one is devoted to the duties of Saturday night, and the other two to recreation.

"During the three summer months the evening reading classes are discontinued, as the patients remain in the pleasure grounds until bedtime.

"The useful and profitable employment of the patients has been carried as far as appeared to be consistent with their sanitary condition."

Appended to the report are several valuable tables, and a highly eulogistic report from the Commissioners in Lunacy, who speak in glowing terms of the condition of the asylum. With deference to the medical officers of county asylums, we question the good taste of publishing these reports of the official visits of the Commissioners; they are not written for publication, and we think should not be ostentatiously paraded in the annual records of the asylum. However, Dr. Bucknill errs, if error it can be called, in good company, and is only adopting the course generally pursued by all the medical superintendents of public asylums.

The "Sixteenth Annual Report of the Suffolk Lunatic Asylum" embodies many interesting particulars. But first, as regards the statistics of the asylum, it appears from Dr. Kirkman's statement that—

"At the close of the last year there were 255 patients in the house; there have been admitted in this year 93; 42 have been discharged cured; 9 have been removed or returned to their friends very much relieved, and 36 have died. The numbers of male and female patients have been nearly equal in the admissions, discharges, and deaths.

| | M. | F. |
|---|---|---|
| Admitted ... ... ... ... | 49 | 44 |
| Discharged ... ... ... ... | 22 | 20 |
| Ditto relieved ... ... | 2 | 7 |
| Died ... ... ... ... | 19 | 17 |

"These numbers show but little variation in any respect from those of the late previous years. The mortality, which was unusually low last year, has been increased by nine this year; an event most fully anticipated, and noted in the report for 1852, from the different stages of great bodily exhaustion, in which many of the aged inmates were received."

The subjoined facts speak volumes in favour of this institution:—

"If the general expressions of contentment amongst the patients are any proof of this healthy feature, we have as conspicuous indices to judge by as any to which we could point at any time. A man after an absence of several years was lately re-admitted; he was a tailor, and directly he entered the house he went up to take possession of his former place of occupancy, and he asked for some work with the expression of satisfaction, 'Well, I'm glad to get home again.' It is this home character that we would endeavour to secure; and lest the paramount importance of this domesticity should ever be lost sight of, it may be well to record it as being the chief object of attainment for successful treatment of the insane. They are almost universally ready to recognise it themselves, and it is a feeling which is very seldom disturbed or broken by the patients in this house.

"The truth of the above was shown in no small degree only a few weeks back, by two out-county patients who left us for Essex. They were both old residents; one having been here twenty-one years, the other twenty-five years. They were much distressed at leaving, the longest resident the most so. She had always looked upon this house as her permanent home, and she would echo the desire of another old patient, most singularly but expressively conveyed, 'that she should have the *privilege* of being buried in the asylum ground.' This attached faithful creature had been for all these years a most valuable assistant in the wash-house and laundry, she was always regularly and willingly employed. Three days in the week in each of these places she worked for twenty-five years, taking little notice of others, unless something *very* provoking excited her displeasure. When she was prevented from going into the laundry on the morning of her expected removal, and when the reason of it was explained to her by signs (for she was very deaf), she looked very sorrowful, and said, 'she would not take any clothes with her, but leave them till she should come back again,' she positively refused to believe that she was going entirely away. It was a 'sorry sight' to witness the removal of this grateful and attached patient: as the carriage came up to the gate she turned very pale, and the tears dropped into her lap, as she took leave of one after another, fondling over them and kissing them. Her industry had procured her some trifling articles of fancy dress, and which always delighted her, but she could not be persuaded to take any of them with her. We have always encouraged this feeling of possession in trifling changes of dress, and we look on it as one of no small importance to gratify."

We have only room for the following account of a remarkable case of attempt at suicide. We would premise that this patient had on several previous occasions endeavoured to accomplish self-destruction. Immediately before admission he had tried to strangle and drown himself:—

"On the afternoon of the 15th the house surgeon was suddenly called to him by his attendant, and he found him suffering from symptoms evidently arising from the existence of some foreign body in the throat, nothing either solid or fluid could be swallowed, there was a choking sensation with sudden spasmodic cough. A probang with an ivory top was introduced into the œsophagus, which rested half-way between the lower part of the pharynx and the cardiac orifice of the stomach upon something *hard*. By careful manipulation this substance was pushed down into the stomach, and now several hard bodies conveying the sensation of *stones* could be distinctly felt with the probang. Upon questioning the man he acknowledged that he had swallowed 200 common gravel stones, with the hope that they would kill him, at the same time he expressed great thankfulness for the relief afforded him and promised never to attempt self-destruction again. By repeated doses of castor-oil a great number of stones were brought away, and all were supposed to have passed safely through. At the end of six days, however, he sent for the house-surgeon again, saying that there was a large stone at the lower end of the bowel, and he could not pass it. On the introduction of the finger, several stones with sharp jagged edges could be distinctly felt impacted in fæcal matter, and the mass appeared to be so large that it seemed impassible without laceration. Whilst an enema was being prepared for him, he suddenly passed a large mass, 8⅝ inches in circumference, and containing seventy-two stones, and many small pieces of brick, &c., the whole weighing nearly 7 ounces. There was no laceration. His bodily health improved after this, but his mind remained unsettled. It was imagined and hoped that this unsuccessful effort, with its painful instruction, would have been his last attempt, but it was not so. He continued with maniacal obstinacy to resist all food, and he was obliged to be fed frequently by the stomach-pump with good beef-tea and wine. On the 11th of August he made another fearful attempt to burn himself to death. He was assisting the attendant in cleaning the galleries, with several other patients about him, when, as if on a sudden impulse, he ran and threw himself into the fire under the bath. He burnt his chest a good deal and the cartilages of his ribs; and for a long while he refused to take any medicine, or to have any application to the burn. He has of late, however, been gradually more yielding, and is now very much better, and recovered entirely from the injury; he will occasionally talk with some degree of cheerfulness, and seems apparently struggling against an almost irresistible impulse. Though this is a very aggravated case (the man literally having tried every element, earth, air, fire, and water, to accomplish his end), it is only one out of the number alluded to before."

This is a type of case met with in most asylums, public and private. Considering the degree of indulgence and liberty extended towards the insane in all well-conducted asylums, as well as the cunning, ingenuity, and cleverness frequently manifested by such patients, we are astonished that accidents of this kind do not more frequently occur.

It appears from the "Sixth Report of the Somerset County Lunatic Asylum for 1853," that "at the termination of 1852 the number of patients remaining in the asylum were 342, being only an increase of 2 on the preceding year. In 1853, the admissions have been 69 males, 64 females, making a total of 133; of these 7 males and 12 females were re-admissions. During the year there were discharged 33 males, 30 females; and died 28 males, 21 females; total at the end of the year, 363."

Dr. Boyd's report is full of interesting matter. The tables are extremely valuable, and must, in their preparation, have entailed upon him great labour. They are well worth the study of all engaged in these abstruse inquiries. The analysis appended to the tabular statements very much enhances their importance.

The last "Annual Report of the Medical Superintendent of the Dorsetshire County Lunatic Asylum" contains a gratifying account of the condition of that institution. It appears "there have been admitted during the year 36 patients, (19 males and 17 females); 14 patients have been discharged, (8 males and 6 females); and 7 have died."

The report contains the usual statistical tables, which appear to be carefully drawn up.

We have before us the "First Annual Report of the Joint Lunatic Asylum for the Counties of Monmouth, Hereford, Brecon, Radnor, and the City of Hereford." This asylum was built for 254 patients, at a cost of 37,083*l.*, including the purchase of a site and all expenses, at a cost of 146*l.* per head. The report says,—"With a probable additional outlay of 1200*l.*, which ought to be immediately undertaken, they will have a building capable of receiving 306 patients, at a cost of 125*l.* per head."

The charge for the "maintenance was, from the opening of the asylum to the 31st December, 1852, 10s. per head per week; since that time it has been 8s. 6d. In consequence of the present high price of provisions the charge must be again raised to 10s."

Dr. Allen presents us with the following statistics of the asylum:—

"A second year has now elapsed since the opening of this asylum for the reception of the insane poor.

"At the close of the year 1852 there remained in the asylum 207 patients, viz., 88 males and 119 females. 93 persons have been admitted during the year 1853, 46 males and 47 females; of these 1 male and 1 female were criminal lunatics, admitted under the warrant of the Secretary of State. There have been 6 re-admissions during the year.

"The discharges during the year have amounted to 42, viz., 23 males and 19 females; of these 22 males and 18 females were recovered or relieved, and 1 male and 1 female were discharged by the desire of friends, but they were not improved.

"The deaths during the year have amounted to 24, viz. 10 males and 14 females. The mortality, it will be observed, among the males has decreased, while that of the females has considerably increased in comparison with that of the previous year—one person died two days, one three days, one eight days, one ten days, one fifteen days, and one a month after admission. With two exceptions, all had well marked symptoms of cerebral disease, which in the majority of cases was verified by post-mortem examinations. The two exceptions were, one a female, who on admission was suffering from acute phthisis complicated with intermittent mania, the other an idiotic girl, who died from inflamed sore throat.

"There now remain in the asylum 234 patients, viz. 101 males and 133 females."

It appears from the last report of the "Littlemore Asylum for 1853," under the medical superintendence of Mr. William Ley, that—

"The number of patients resident, at the close of the year 1853, was 28 more than at its opening. It had risen from 366 to 406, and had again been reduced to 394. The admissions in the year (including the readmission of three patients within twelve months of their discharge, and eleven after longer periods,) were barely less than in preceding years, being 107; they were in 1852, 109; the average number of patients sent by their parishes in five years was 110 in each. The recoveries (39 in number) were equal to those of the previous year; and the mortality (namely, 34 deaths) was diminished."

The report contains the usual number of tables and a full detail of the yearly expenditure of the asylum. The former we cannot transfer to our pages, and the latter has only a local interest.

The "Report of the Gloucester County Lunatic Asylum for 1853" contains little else than numerous tabular statements. Dr. Williams has made no special report; of course the statistical tables are drawn up by himself, and for these he is entitled to credit. The following information we extract from the report:—

| | 1st Class. | | 2nd Class. | | 3rd Class. | | | | Total. |
|---|---|---|---|---|---|---|---|---|---|
| | M. | F. | M. | F. | M. | F. | M. | F. | |
| Remaining in the house, Dec. 31, 1852 ... ... ... ... | 9 | 6 | 15 | 14 | 115 | 151 | 139 | 171 | 310 |
| Admitted during the year ... | 5 | 1 | 4 | 7 | 56 | 55 | 65 | 63 | 128 |
| Re-admitted ... ... ... ... | 2 | ... | 2 | 1 | 10 | 9 | 14 | 10 | 24 |
| Chronic cases re-admitted from Fairford ... ... ... | ... | ... | ... | ... | ... | 21 | ... | 21 | 21 |
| Total under treatment during the year ... ... ... ... | 16 | 7 | 21 | 22 | 181 | 236 | 218 | 265 | 483 |
| Discharged— | | | | | | | | | |
| Recovered and gone ... | 3 | ... | 9 | 4 | 16 | 23 | 28 | 27 | 55 |
| Out on trial ... ... ... | ... | ... | ... | ... | 1 | ... | 1 | ... | 1 |
| Relieved ... ... ... | 2 | ... | 1 | ... | 5 | 2 | 8 | 2 | 10 |
| Not relieved, removed by friends ... ... ... | ... | ... | 1 | 1 | ... | 1 | 1 | 2 | 3 |
| Died ... ... ... ... ... ... | ... | ... | 1 | 1 | 24 | 14 | 25 | 15 | 40 |
| Total ... ... | 5 | ... | 12 | 6 | 46 | 40 | 63 | 46 | 109 |
| Remaining in the house, Dec. 31, 1853 ... ... ... ... | 11 | 7 | 9 | 16 | 135 | 196 | 155 | 219 | 374 |

Average number in the House each week in the year 355
Ditto attending Chapel (two Services) ... 289
Ditto daily employed ... ... ... ... 216

| | | | |
|---|---|---|---|
| Total number admitted from opening of Institution in 1823 ... ... | | | 2871 |
| Ditto | discharged ... ... ... ... | ditto ... ... ... | 2497 |
| Ditto | recovered ... ... ... ... ... | ditto ... ... ... | 1454 |
| Ditto | relieved ... ... ... ... ... | ditto ... ... ... | 183 |
| Ditto | removed or transferred ... ... | ditto ... ... ... | 253 |
| Ditto | discharged, harmless or improper, | ditto ... ... ... | 73 |
| Ditto | died ... ... ... ... ... ... ... | ditto ... ... ... | 533 |
| Ditto | remaining on the Books ... ... ... ... ... ... ... | | 375 |
| Out on trial ... ... ... ... ... ... ... ... ... ... ... ... ... ... | | | 1 |
| Remaining in the House, Dec. 31, 1853 ... ... ... ... ... ... ... | | | 370" |

According to the "Fortieth Report of the Staffordshire Lunatic Asylum," under the medical superintendence of Mr. Wilkes, there were, at the termination of the year 1852,—

"Four hundred patients in the asylum—viz., 212 males and 188 females. During the year 1853, 91 males and 81 females were admitted, making a total of 572 patients under treatment. Of these, 43 males and 53 females have been discharged recovered; 11 males and 8 females relieved or incurable; and 34 males and 24 females have died; leaving on the 31st December, 399 patients in the house, of whom 215 were males and 184 females.

"The average number of patients resident throughout the year was 405.

"The admissions have slightly exceeded those of the previous year, being respectively 172 and 166. Of these 107 may be termed recent cases, the disorder being reported to have existed for various periods not exceeding six months, whereas in the previous year only 87 were of this class. Fifteen were stated to have been insane from 6 to 12 months, and 50 for much longer periods; consequently nearly the whole of these must be regarded as chronic and incurable cases, and a permanent burden to the rate-payers."

It appears from the "Report of the Physician of St. Luke's Hospital for 1853," that, during the preceding year,—

"Fifty-eight male and 117 female patients have been admitted, there having remained from the previous year 26 males and 63 females, under treatment, making together 84 males and 180 females; of these 37 men and 82 women have been discharged cured, 12 men and 28 women uncured, and 6 men and 8 women have died; 5 men and 12 women have been removed at the request of friends. The total number therefore of patients discharged cured is 119, uncured 40, deaths 14; giving a per centage of 68·79 cured, of 23·12 uncured, and of 8·09 deaths.

"The per centage of recoveries has been higher this year than in any previous year, except those of 1842 (when it was 70·37) and 1851 (when it was 74·01)."

The physicians (Drs. Sutherland and Philp) report favourably of the sanitary state of the hospital. They recommend to the governors the establishment of a branch asylum, and propose that a farm should be purchased in the neighbourhood of London, for the reception of boarders and convalescent patients. We hope the governors will take this excellent suggestion into immediate consideration. Eight cases of recovery are referred to in which the insanity had existed for from five to twelve years!

The "First Annual Report of the Medical Officers of the Norfolk County Asylum" is evidently drawn up with great care by, we presume, the late resident physician, Dr. Foote, who was, we think, so unfairly dismissed from the institution. The subjoined facts will illustrate the statistics of the asylum:—

"During the past 12 months, 83 patients have been admitted—viz., 36 males and 47 females.

"On the 31st December, 1852, there were in the asylum 139 males and 159 females; total, 298. The whole number under treatment during the year has been 381; the average number daily resident, 304·91, or 139·66 males and 165·25 females.

"The number of deaths has been 36, or 19 males and 17 females; and the number of recoveries 38, or 16 males and 22 females; and the number discharged, not cured, has been 6—viz., 2 males and 4 females."

The "Report of the Lunatic Asylum at Rainhill for 1853" contains no extractable matter likely to prove interesting to our readers, with the exception of the subjoined table:—

ADMISSIONS AND DISCHARGES DURING THE YEAR 1853.

| | M. | F. | Total. | M. | F. | Total. |
|---|---|---|---|---|---|---|
| Remaining in the Asylum, Jan. 1, 1853 ... ... ... ... ... ... | 170 | 204 | 375 | | | |
| Admitted during the year ... ... | 75 | 70 | 145 | | | |
| | | | | 245 | 274 | 519 |
| Discharged recovered ... ... ... | 29 | 23 | 52 | | | |
| Ditto improved ... ... ... | 1 | 1 | 2 | | | |
| Ditto unimproved ... ... | 1 | 4 | 5 | | | |
| Escaped ... ... ... ... ... ... | 2 | 1 | 3 | | | |
| Died ... ... ... ... ... ... ... | 31 | 28 | 59 | | | |
| | | | | 64 | 57 | 121 |
| Remaining in the Asylum, Jan. 1, 1854 ... ... ... ... ... ... | ... | ... | ... | 181 | 217 | 398 |
| Per centage of recoveries on the numbers under treatment ... | ... | ... | ... | ... | ... | 10 |
| Per centage of deaths on the numbers under treatment ... ... | ... | ... | ... | ... | ... | 11·30 |
| Per centage of recoveries on admissions during the year ... ... | ... | ... | ... | ... | ... | 35·85 |
| Average number resident during the year ... ... ... ... ... | ... | ... | ... | ... | ... | 394 |

Having briefly analyzed the reports of the English asylums, we now proceed to consider the Scotch institutions for the insane. The

"Fortieth Annual Report of the Glasgow Royal Lunatic Asylum for 1853," under the able management of Dr. Mackintosh, the resident physician, is of peculiar interest. In the preliminary portion of the report, Dr. Mackintosh refers to the remarkable increase of cases of lunacy in the west of Scotland, by which all the accommodation, good, bad, and indifferent, has been called into requisition. It appears that—

"The number of patients admitted during the year was 319, being 53 more than last year. And the increase would have been greater had it not been found necessary to cease admitting patients for the reason already specified—viz., the want of room. So great appears to be the increase of lunacy in this part of the country, that though a very great number of patients have been taken to parochial receptacles whenever the parish was of sufficient population to admit of having a poor's house, the numbers in this asylum have always, more or less, tended to increase."

When attempting to account for this increase of cases of insanity, Dr. Mackintosh observes,—

"The more immediate cause of the increase of lunacy is to be sought for rather in the social condition of the time in which we live. Ours is a time of great mental activity and excitement. Men's minds are constantly on the stretch. Nor is this state of things confined to the higher and more opulent classes of society. Among the great mass of the labouring population during the past year, there has been much commotion and excitement, manifesting itself particularly in the shape of 'strikes,' and the like. There has been, if not war, at least rumours of war. In the increase of population, and the excitement of the times, in the varied mental emotions to which passing events have given birth, and probably to the increase of intemperance, do we look for the more immediate cause of the increase of lunacy which has characterized the past and some of the preceding years."

According to the statistics of the asylum, it appears that—

"The cases of mania exceed those of monomania, including melancholia; that the number of males exceeds that of females; and that the ratio of melancholia to mania is much higher in females than in males."

The number of married and unmarried patients were nearly equal. The married and widowed together considerably exceed the number of those unmarried. The ages of the patients—

"Range between 20 and 90. There were none under 20 years of age. Between the ages of 30 and 50, or in the prime of life, it is found that there were by far the greatest number of patients."

It appears that the cases of insanity from intemperance were in a ratio of 1 to 5 in the whole number of patients admitted. Hereditary predisposition appears to have been a fruitful cause of insanity. It is observed that—

"It sometimes happens that two, three, or even four members of the same family are confined in asylums at the same time; and there are some families who have at least one member constantly in confinement. From the unwillingness which the friends of patients manifest to reveal the circumstance of the previous existence of insanity in the family, whether in the direct line or in lateral branches, the above can only be considered as an approximate estimate of the numbers of those in whom the malady is hereditary."

Dr. Mackintosh says that—

"In those admitted during the past year, we find that the physical causes very much exceed both the moral and mental ones combined, a result which does not coincide with the speculations of many eminent men on this subject, and which may, according to M. Guislain, arise from insufficiency and incorrectness of investigation, or the want of close personal intimacy with patients. As to the latter circumstance, it is to be remarked, that a very great number are either unable or unwilling to give correct information on the subject, and that the information which many communicate is found to be manifestly and totally at variance with the truth. As to the insufficiency of investigation which is supposed to be inevitable, in the inquiries of physicians connected with large public institutions, this may be true to a certain extent, as regards the minute psychological analysis, which is presumed to be necessary to the discovery of the moral causes, by which, in many cases, the disease has been produced."

This physician concludes, from the table recording the occupations of those who were admitted, that one occupation does not predispose more than another to insanity. He says, "The active or sedentary, the mental or physical nature of the occupation does not seem to exercise any particular influence in the production of mental disorder."

One hundred and sixteen patients were discharged as cured. Out of this number—

"Thirty-eight males and 57 females were cases of mania; 3 males and 16 females were cases of monomania; and one male and one female were cases of dementia;—in all 42 males and 74 females.

"Of the whole, 50 or about one-half were less than a month ill previous to admission, showing what all statistics prove, that recovery is most likely to occur if the patient is put early under treatment; that the probability of recovery becomes less and less according to the length of time during which the patient has been ill previous to admission till all reasonable hope disappears."

When speaking of the social treatment of the insane, Dr. Mackintosh makes some sensible remarks:—

"To many who are convalescent, the restraint of an asylum becomes irksome in the extreme; but when there is a large number freely associating with one another, and meeting daily—in the library, billiard-room, or drawing-room, in the bowling-green, and in the grounds of the asylum—friendships are formed and feelings are excited of a wholesome and salutary kind; so much so, indeed, that not unfrequently, however strange it may appear, it happens that some leave the asylum with feelings rather of regret than pleasure. And though such feelings are considerably modified by return to the active business of life, they delight to revisit and correspond with their less happy friends in affliction."

We regret that the pressure of other matter upon our space deprives us of the pleasure of quoting more at length from this excellent and interesting document. This asylum, we can report from personal examination, is in capital order, and reflects great credit upon the talented physician who presides over it. Dr. Skae's "Report of the Royal Edinburgh Asylum for 1853" is, like the preceding one, replete with valuable and interesting matter.

The subjoined table gives the general statistics of the year :—

| | Males. | Females. | Total. |
|---|---|---|---|
| Number of inmates at the close of 1852... | 275 | 268 | 543 |
| Admitted during the year 1853 ... ... | 103 | 133 | 236 |
| Total number under treatment ... | 378 | 401 | 779 |
| M. F. T. | | | |
| Discharged...79 78=157 | | | |
| M. F. T. | | | |
| Of whom were cured ... 58 50=108 | | | |
| ,, ,, uncured... 21 28= 49 | | | |
| Died ... ... 36 41= 77 | 115 | 119 | 234 |
| Total number at the close of 1853 ... ... | 263 | 282 | 545 |

Average number daily resident during the year 1853.

| Males. | Females. | Total. |
|---|---|---|
| 273 $\frac{134}{365}$ | 280 $\frac{30}{365}$ | 553 $\frac{164}{365}$ |

When speaking of the causes of insanity, Dr. Skae remarks :—

"Of the males, two were caused by imprisonment; in the one, the disease was suddenly developed by the shock experienced at being imprisoned on a charge of theft, of which the lad continued to protest his entire innocence; and, in the other, the insanity seemed to have developed itself under the peculiarities of prison discipline acting upon a mind naturally weak. In another, the terrors incident to having been left alone in a house with the dead body of his master; and in another the anxiety, and fatigue, and grief of nursing a young gentleman who died, appear to have operated in the development of the malady. Two of the young men had been abroad; the one in America, the other in Australia; and in both the novelty, excitement, and mode of living, are supposed to have been the principal causes of the insanity. Of the females, three became insane as the sequence of early marriages, for the anxieties and responsibilities of which they were incapacitated, partly by natural deficiency, and partly from deficient education. Three were servants, brought up in the innocence and seclusion of pious homes in the remote north, and suddenly exposed to the worry, and exactions, and temptations attendant upon service in metropolitan houses."

We quote *in extenso* Dr. Skae's account of the *post mortem* examinations made in 61 cases:—

"Of those examined, 3 had been cases of acute, 2 chronic, and 1 periodic mania, 23 dementia, 3 dementia with epilepsy, 1 mania with epilepsy, 1 melancholia, 1 moral insanity, 1 delirium tremens, 11 monomania, 12 general paralysis, 2 congenital imbecility.

"Calvarium was of unusual thickness in 32 cases: 1 of chronic mania, 1 of acute dementia, 13 of dementia, 1 of dementia with epilepsy, 5 of general paralysis, 8 of monomania, 1 of moral insanity, and 2 of congenital imbecility.

"Calvarium was thinner than usual in 13 cases: 3 of acute mania, 1 of chronic mania, 1 of melancholia, 3 of dementia, 2 of monomania, and 3 of general paralysis.

"Diploe was absent in 14 cases: 1 of acute mania, 1 of acute dementia, 6 of dementia, 3 of monomania, 1 of moral insanity, 1 of general paralysis, 1 of congenital imbecility.

"Elongation of anterior clinoid process on right side was observed in one case of mania with epilepsy.

"Ætheromatous deposit in arteries of brain was found to a great extent in one case of general paralysis.

"Increased thickness of dura mater was found in 18 cases: 1 of acute mania, 1 of moral insanity, 4 of dementia, 2 of dementia with epilepsy, 3 of monomania, 6 of general paralysis, and 1 of congenital imbecility.

"Thinness of dura mater was noticed in 5 cases: 3 of dementia, 1 of monomania, and 1 of general paralysis.

"Ossific deposit in falx cerebri existed in 2 cases: 1 of dementia, and 1 of monomania.

"Adhesion of dura mater to calvarium existed in 17 cases: 3 of acute mania, 1 of chronic mania, 1 of melancholia, 3 of dementia, 1 of dementia with epilepsy, 5 of monomania, 1 of moral insanity, 1 of general paralysis, and 1 of congenital imbecility.

"Opacity and thickening of arachnoid was found in 39 cases: 2 of acute mania, 1 of periodic mania, 1 of chronic mania, 1 of acute dementia, 9 of dementia, 1 of dementia with epilepsy, 1 of melancholia, 7 of monomania, 2 of moral insanity, 12 of general paralysis, and 2 of congenital imbecility.

"Crystalline-like deposit over general surface of arachnoid was noticed in two cases: 1 of dementia, and 1 of general paralysis.

"Congestion of membranes was noticed in 19 cases: 2 of acute mania, 1 of periodic mania, 1 of chronic mania, 7 of dementia, 1 of dementia with epilepsy, 2 of monomania, 4 of general paralysis, and 1 of congenital imbecility.

"Adhesion of membranes to cortical substance was found in 10 of general paralysis.

"Serous effusion into sac of arachnoid existed in 53 cases: 3 of acute mania, 1 of periodic mania, 1 of chronic mania, 1 of acute dementia, 18 of dementia, 2 of dementia with epilepsy, 1 of mania with epilepsy, 11 of monomania, 2 of moral insanity, 11 of general paralysis, and 2 of congenital imbecility.

"Sub-arachnoid serous effusion was found in 47 cases: 3 of acute mania, 2 of chronic mania, 1 of periodic mania, 1 of acute dementia, 17 of dementia, 2 of dementia with epilepsy, 10 of monomania, 2 of moral insanity, and 9 of general paralysis.

"Sero-sanguinolent effusion into sac of arachnoid was found in 5 cases: 1 of chronic mania, 2 of dementia, 1 of dementia with epilepsy, and 1 of general paralysis.

"Sub-arachnoid sero-sanguinolent effusion occurred in 2 cases: 1 of mania with epilepsy, and one of dementia.

"The convolutions of cerebrum were noticed to be remarkably diminished in size in 4 cases: 1 of dementia, 1 of epilepsy with dementia, 1 of general paralysis, and 1 of congenital imbecility.

"Wasting of optic nerves and commissure occurred in one case of general paralysis.

"Paleness of the grey matter was noticed in 24 cases: 1 of acute mania, 1 of periodic mania, 1 of melancholia, 13 of dementia, 3 of monomania, 1 of moral insanity, 3 of general paralysis, and 1 of congenital imbecility.

"Grey matter was of a dark tint in 10 cases: 1 of mania with epilepsy, 1 of dementia, 2 of monomania, 6 of general paralysis.

"Grey matter of a violaceous tinge in 3 cases: 1 of chronic mania, and 2 of general paralysis.

"Grey matter was softened in 31 cases: 1 of acute mania, 2 of chronic mania, 1 of acute dementia, 10 of dementia, 5 of monomania, 1 of moral insanity, and 11 of general paralysis.

"White matter was softened in 16 cases: 7 of dementia, 1 of dementia with epilepsy, 5 of monomania, 3 of general paralysis.

"Serous effusion into lateral ventricles was found in 37 cases: 2 of acute mania, 1 of chronic mania, 1 of melancholia, 11 of dementia, 2 of dementia with epilepsy, 6 of monomania, 2 of moral insanity, 11 of general paralysis, and 1 of congenital imbecility.

"Sero-sanguinolent effusion into lateral ventricles occurred in 2 cases: 1 of acute mania, and 1 of mania with epilepsy.

"Foramen of Monro unusually large in 15 cases: 1 of acute mania, 1 of chronic mania, 3 of dementia, 1 of dementia with epilepsy, 4 of monomania, 1 of moral insanity, 3 of general paralysis, and 1 of congenital imbecility.

"Crystalline-like deposit in membrane of lateral ventricles was found in 8 cases: 1 of acute mania, 1 of chronic mania, and 6 of general paralysis.

"Cystic bodies in choroid plexuses occurred in 27 cases: 2 of acute mania, 1 of chronic mania, 1 of periodic mania, 8 of dementia, 1 of epilepsy with dementia, 1 of acute dementia, 6 of monomania, and 7 of general paralysis.

"Disorganisation of the right corpus striatum was found in a case of monomania.

"Pineal body was unusually large in 4 cases: 1 of chronic mania, 1 of mania with epilepsy, 2 of monomania.

"Absence of grit in pineal body in 19 cases: 1 of acute mania, 1 of chronic mania, 1 of mania with epilepsy, 1 of acute dementia, 6 of dementia, 1 of melancholia, 2 of monomania, 1 of moral insanity, 4 of general paralysis, and 1 of congenital imbecility."

On the question of medical treatment Dr. Skae says:—

"In regard to treatment, I may repeat in general terms, that I have continued to derive the greatest amount of benefit in acute and recent cases, from the employment of the prolonged warm bath, accompanied by cold affusion on the head, in some instances the effects being almost sudden, and in not a few very rapid and permanent. The judicious use of opiates in another class of cases, and the removal, by appropriate remedies, of local affections in others, are the next sources from which the greatest amount of benefit from medical treatment has been derived. In a very large class of cases brought to the institution, the disease has supervened in persons of a scrofulous and feeble constitution, upon habits of over-exertion, combined often with insufficient nourishment, poverty, and anxiety; and in these a generous diet and a moderate allowance of stimulants have been found of great efficacy in the removal of the disease. The beneficial influence of a liberal diet, and a liberal allowance of malt liqours in the treatment of the insane, has been fully proved by the statistics of the various asylums throughout the empire, the proportion of recoveries bearing a very remarkable relation to the dietary and the amount of malt liquor comprised in it.

"In the treatment of patients, on the other hand, whose disease has been brought on by the excessive use of whisky, wine, opium, and other stimulants, I have not found in those cases where it has been adopted, any bad effect to result from the sudden and total cessation of their use, but, on the contrary, it appears to be the method ultimately most agreeable to the patients themselves, the complete suspension of the stimulants being followed within a very short time by a complete absence of the craving for them."

The "Thirty-fourth Annual Report of the Dundee Royal Asylum for 1854" contains the following statistics:—

"At the date of the last report there were 201 patients remaining in the asylum: there have been 41 admissions since—making the total number of patients during the year 242. Of these 25 have been discharged—19 of them cured, 4 improved, and 2 by desire; and 12 have been removed by death. The total number of patients, therefore, in the institution is 205. The daily average number throughout the year has been 204."

We are compelled for want of space to postpone our prepared analyses of the Perth, Kilkenny, Belfast, and North Wales Asylums. They all contain valuable statistical and general information to which we shall more particularly refer when analyzing their contents. Dr. Boisrogon's excellent "Report of the Cornwall County Lunatic Asylum" will also be noticed in our next number.

